# Informing immunotherapy with multi-omics driven machine learning

**DOI:** 10.1038/s41746-024-01043-6

**Published:** 2024-03-14

**Authors:** Yawei Li, Xin Wu, Deyu Fang, Yuan Luo

**Affiliations:** 1 Department of Preventive Medicine, Northwestern University, Feinberg School of Medicine, Chicago, IL 60611 USA; 2 Center for Collaborative AI in Healthcare, Northwestern University, Feinberg School of Medicine, Chicago, IL 60611 USA; 3 Department of Medicine, University of Illinois at Chicago, Chicago, IL 60612 USA; 4 Department of Pathology, Northwestern University Feinberg School of Medicine, Chicago, IL 60611 USA

**Keywords:** Cancer models, Computational models, Cancer, Cancer genomics, Tumour immunology

## Abstract

Progress in sequencing technologies and clinical experiments has revolutionized immunotherapy on solid and hematologic malignancies. However, the benefits of immunotherapy are limited to specific patient subsets, posing challenges for broader application. To improve its effectiveness, identifying biomarkers that can predict patient response is crucial. Machine learning (ML) play a pivotal role in harnessing multi-omic cancer datasets and unlocking new insights into immunotherapy. This review provides an overview of cutting-edge ML models applied in omics data for immunotherapy analysis, including immunotherapy response prediction and immunotherapy-relevant tumor microenvironment identification. We elucidate how ML leverages diverse data types to identify significant biomarkers, enhance our understanding of immunotherapy mechanisms, and optimize decision-making process. Additionally, we discuss current limitations and challenges of ML in this rapidly evolving field. Finally, we outline future directions aimed at overcoming these barriers and improving the efficiency of ML in immunotherapy research.

## Introduction

The immune system is crucial in monitoring cancer and identifying neoantigens produced by tumor cells that can trigger cellular immune responses^1^. But tumor cells have developed strategies to evade immune surveillance^2^. To address this, cancer immunotherapy was developed, aiming to stimulate the immune system or create lab-engineered substances that restore the ability to recognize and eliminate cancer cells. Immunotherapy options include immune checkpoint inhibitors (ICI), cancer vaccines, adoptive cellular therapies (ACT), cytokine, tumor-infecting viruses, targeted antibodies, and adjuvants. While immunotherapy has significantly improved patient outcomes, its effectiveness is confined to a small and unpredictable subset of patients within a given cancer diagnosis^3^, and immune-related adverse events (irAEs) may occur^4^. Therefore, precise identification of a patient’s tumor microenvironment (TME) and of the ability to predict its immunotherapy response are essential for enhancing overall immunotherapy effectiveness.

Current prediction of immunotherapy response relies on biomarkers such as immune-cell infiltration^5^, tumor mutational burden (TMB)^6^, PD-1/PD-L1 expression^7^, CTLA-4 expression^8^, mismatch repair (MMR) and microsatellite instability (MSI)^9^. However, existing clinical practices based on simplistic threshold-based methods lack accuracy. In this context, machine learning (ML) technologies have emerged as valuable tools, offering the potential to refine the precision of immunotherapy response prediction. By harnessing sophisticated algorithms and analyzing extensive datasets, ML models can discern intricate patterns and interactions among various molecular biomarkers, providing a more nuanced understanding of the complex immunotherapy tumor microenvironment. These state-of-the-art ML models not only capture subtle relationships between individual biomarkers but also adapt to the dynamic nature of immune responses, offering a more comprehensive and adaptable approach than traditional threshold-based methods. Indeed, ML-based approaches have shown capacity in various oncology applications, including early diagnosis^10^, cancer type classification^11^, the complexity and plasticity of TME and immune system deciphering^12^, response and prognosis prediction^13^, and potential neoantigen detection^14^.

**Fig. 1:**
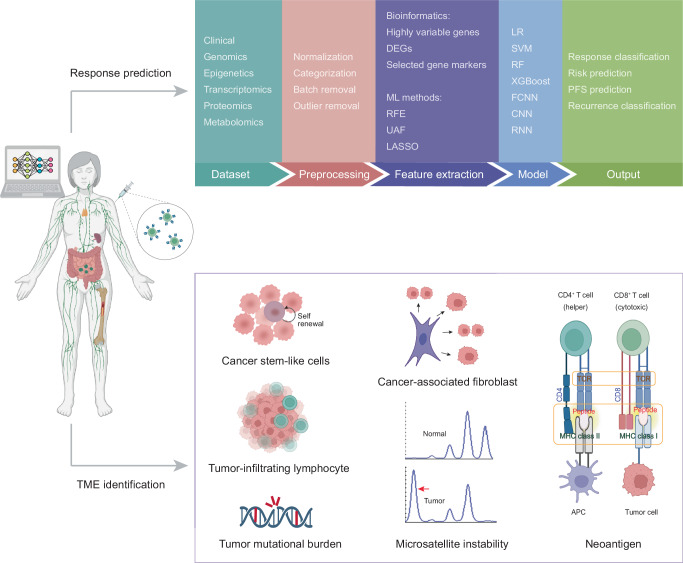
Genomic landscape of machine learning in tumor immunotherapy. We provide an overview of machine learning methodologies applicable to different aspects of tumor immunotherapy including prediction of immunotherapy responses, identification of response-associated biomarkers, and analysis of the TME. ML machine learning, DEG differentially expressed gene, RFE recursive feature elimination, UAF univariate association filtering, LASSO the least absolute shrinkage and selection operator, LR logistic regression, SVM support vector machine, RF random forest, FCNN fully-connected neural network, CNN convolutional neural network, RNN recurrent neural network, PFS progression-free survival, TME tumor microenvironment.

This review summarizes the application of ML in molecular analyses related to immunotherapy, including prediction of immunotherapy responses, identification of response-associated biomarkers, and analysis of the TME (Fig. 1). It also explores ML approaches developed to optimize the identification of crucial neoantigens in personalized immunotherapy. Additionally, this review discusses the challenges and opportunities encountered in current research endeavors, aiming to enhance understanding and recognition of the significant contribution of ML in advancing cancer immunotherapy.

## Employing machine learning for predicting immunotherapy response and identifying biomarkers associated with response

While immunotherapy has brought significant benefits to cancer treatments, its effectiveness remains confined to a small and unpredictable subset of patients with a given cancer diagnosis^15^. Moreover, the treatment process often imposes substantial financial, physical and mental care burden on patients. Acknowledging these challenges, researchers are increasingly directing their efforts toward identifying valuable molecular biomarkers capable of predicting immunotherapy outcomes and improving its overall utility^16^. Considering the complex omics space, conducting extensive sampling through experimental methods is impractical. Consequently, in silico approaches, including those employing ML algorithms, provide an opportunity to address this critical need.

**Fig. 2:**
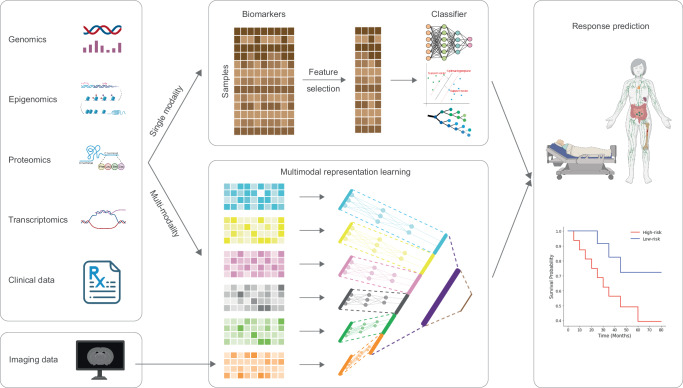
An overview of machine learning techniques for immunotherapy response prediction. Advancements in sequencing technologies have paved the way for exploring diverse approaches in immunotherapy response prediction. To improve efficiency and mitigate overfitting, dimensionality reduction and feature selection techniques are performed prior to model training. Multimodal models offer the flexibility to train on data with multiple modalities. These models first utilize sub-models to extract unimodal features from each data modality. Subsequently, a data fusion step transforms each extracted unimodal feature into a compact multimodal representation. Finally, a classification sub-model is implemented to infer response based on the integrated features.

Tumors are caused by the accumulation of various genetic variations that regulate the way cells growth and multiplication^17–19^. In light of this, recent studies have turned on ML models to predict a patient’s response to immunotherapy by leveraging his genomic biomarkers and clinical features (Table 1, Fig. 2). Somatic mutations, including single-nucleotide variants (SNVs), insertions, and deletions, provide direct evidence documenting the driving forces behind tumorigenesis and tumor cell proliferation^20^. These mutations have demonstrated their ability in predicting immunotherapy responses. For example, Peng et al.^21^ used SNV data and convolutional neural network (CNN) model to classify anti-PD-1/PD-L1 therapy response from metastatic non-small-cell lung cancer (NSCLC) patients. Nonsynonymous mutations can alter transcription, subsequently impacting pathway activations and gene functions. Leveraging the distinct changes in gene expression levels, particularly in oncogenes and tumor suppressor genes, ML models can subtly predict immunotherapy response. According to our survey, RNA-based features, including bulk RNA sequencing^22–31^, single cell RNA sequencing (scRNA-seq)^32,33^, flow cytometry^34^, and circulating cell-free microRNA sequencing^35,36^, have been widely implemented in immunotherapy response prediction. Furthermore, the availability of numerous accessible RNA-seq datasets, coupled with the outstanding performance of models utilizing RNA-seq data, has been instrumental in advancing research. From RNA-seq data, many advanced features can be extracted from bioinformatic or ML tools to better characterize tumor genomic profiles, such as tumor-infiltrating lymphocytes (TILs)^32,33^, pathway activity^28^ and cell-cell communication^23^. It is worth noting that some studies have leveraged these features to extract high-level features, thereby enhancing predictive performance. For instance, Wang et al.^22^ utilized TMB information based on SNV data, gene expression information, and support vector machines-recursive feature elimination (SVM-RFE) algorithm to select gene features. Subsequently, they used least absolute shrinkage and selection operator (LASSO) logistic regression classifier to predict responses of urothelial carcinoma patients treated with the PD-L1 inhibitor atzolizumab using selected gene features. Their approach achieved an AUC of 93% in the test dataset. Additionally, they utilized generalized linear models (GLMs) to derive a TMB-related LASSO score (TLS) from the LASSO regression results. The TLS can serve as an effective indicator for immunotherapy response prediction like TMB. Lapuente-Santana et al.^23^ employed regularized multi-task linear regression (RMTLR) to identify interpretable biomarkers in relation to immune cells markers, intracellular networks, and intercellular networks for predicting immunotherapy response. On the other hand, Zeng et al.^28^ implemented a joint nonnegative matrix factorization (NMF) to decompose gene expression matrix, molecular phenotype matrix, and immunotherapy response matrix. This approach aims to identify pivotal genes correlated with immunotherapy response. Similar to RNA-seq data, Shang et al.^37^ and Filipski et al.^38^ have successfully employed DNA methylation profiles (CpG sites) for ICI response prediction in NSCLC^37^ and metastatic melanoma^38^ patients. Apart from these conventional biomarkers, clinical information^39^ and Raman spectroscopy data^40^ have shown promise as reliable biomarkers of ICI response prediction. In a separate study, Sidhom et al.^41^ integrated human leukocyte antigen (HLA) and T cell receptor (TCR) sequencing to predict ICI response in melanoma. Their approach involved employing a multiple-instance learning model that incorporated HLA into the featurization of the TCR sequences to provide a representation of a joint TCR-HLA antigenic latent space. The contextualization of TCR-HLA was then trained on multihead attention networks to learn the attention weights, which were used to predict the final immunotherapy response.

**Table 1:** Publications relevant to machine learning in immunotherapy response prediction

Task	ML Model	ML-based biomarker	Cancer	Patients	Therapy	Validation method	Performance	Input	Output	Ref
Predict response	RF *, CNN	Yes	NSCLC	915	Anti-PD-(L)1	5-fold cross-validation	AUC (0.96–0.97)	55 SNV locations	Response prediction (DCB, PFS, OS)	^21^
Predict response	SVM-RFE *, LASSO regularized LR	Yes	Metastatic BLCA	272	Anti-PD-L1	10-fold cross-validation	AUC (0.93)	TMB related genes	Responder vs. Non-responder and selected genes	^22^
Predict response	Multi-task linear regression using elastic net regularization	No	SKCM, STAD, BLCA, GBM	432	Anti-PD-(L)1	Hold-out	AUC (0.79–0.84)	RNA-based features	Responder vs. Non-responder	^23^
Predict PFS	linear SVM *	Yes	Metastatic gastrointestinal cancer	96	Anti-PD-(L)1	13-fold cross-validation	AUC (0.74)	RNA of 395 genes	DCB vs. non-DCB	^24^
Predict response	SVM and XGBoost	No	Pan-cancer	Not mentioned	ICI	Hold-out	Accuracy (0.88)	RNA of 2387 genes	Responder vs. Non-responder	^25^
Predict response	SVM-RFE *, RF	Yes	SKCM	212	Anti-PD-1	10-fold cross-validation	AUC (0.71–0.87)	RNA + SNV + clinical features	Response prediction	^26^
Predict response	LR *, NN	Yes	Esophageal adenocarcinoma	76	ICI	Hold-out	AUC (0.88–1.00)	RNA	Responder vs. Non-responder and selected genes	^27^
Predict response	A joint NMF-based model *	Yes	Pan-cancer (12 cancer types)	764	Anti-PD-1, anti-PD-L1, anti-PD-L2, anti-CTLA4	5-fold cross-validation	AUC (0.74)	RNA	Responder vs. Non-responder	^28^
Predict response	LASSO regression *,SVM	Yes	NSCLC	122	Anti-PD-(L)1	Hold-out	Significant hazard ratio differences	RNA	Responder vs. Non-responder	^29^
Predict response	LASSO regression *	Yes	NSCLC, UC, RCC	366	Anti-PD-L1	5-fold cross-validation	AUC (up to 0.62)	RNA	Responder vs. Non-responder selected gene features	^30^
Predict response	KNN, Linear SVM, RBF-SVM, GP, RF, DT, NN, AdaBoost, NB, quadratic classifier	No	BCC	11	Anti-PD-1	5-fold cross-validation	Accuracy (0.61–0.97 from different models)	Top 2,000 highly variable genes of CD8 T cell scRNA-seq data	Responder vs. Non-responder	^32^
Predict response	NN *	Yes	SKCM, BCC	43	Anti-PD-1	LOOCV	Accuracy (up to 1.00)	scRNA-seq data of CD8 + T cell	Responder vs. Non-responder	^33^
Predict response	LR	No	GEA	Not mentioned	Anti-PD-1 along with radiation therapy	10-fold cross-validation	Accuracy (up to 1.00)	Expression of selected genes from PMBC	Responder vs. Non-responder	^34^
Predict response	RF	No	NSCLC	213	Anti-PD-1	5-fold cross-validation	AUC (0.76–0.83)	Circulating miRNA + clinical information	Responder vs. Non-responder	^35^
Predict response and identify response related cfmiR biomarkers	RF *	Yes	Metastatic melanoma	47	ICI	Not mentioned	Not mentioned	162 differentially expressed cfmiRs	Responder vs. Non-responder and selected cfmiRs	^36^
Predict response	LASSO regression	No	NSCLC	78	Anti-PD-(L)1	10-fold cross-validation	AUC (0.80)	Differentially methylated CpG sites	Responder vs. Non-responder	^37^
Predict response	LASSO regularized LR	No	Metastatic melanoma	65	ICI	10-fold cross-validation	AUC (0.96)	5000 most variable methylated CpG sites	Responder vs. Non-responder	^38^
Predict response	NN	No	HNSCC	37 + simulated patients	Anti–PD-1	10-fold cross-validation	AUC (0.61–0.90)	Clinical features	Responder vs. Non-responder	^39^
Predict response	RF *,SVM	Yes	Colorectal cancer	25 (mice)	Anti-mouse CTLA4, anti-mouse PD-L1	LOOCV	Not directly showed	Spectra features from Raman spectroscopy	Responder vs. Non-responder and feature contributions	^40^
Predict response	MIL + DeepTCR	No	Not mentioned	43	ICI	Monte Carlo cross-validation	AUC (0.86)	TCR sequencing data + MHC sequencing data	Responder vs. Non-responder	^41^
Predict response	RF	No	Pan-cancer (16 cancer types)	1,479	ICI	5-fold cross-validation	AUC (up to 0.85)	Genomic features based on DNA variants, RNA, demographic and clinical data	Responder vs. Non-responder	^42^
Predict response	SVM, NB, RF, KNN, AdaBoost, boosted LR	No	RCC, UC, SKCM, GBM, BCC	955	Anti-PD-(L)1, anti-CTLA4, anti-PD-(L)1 plus anti-CTLA-4 combination	5-fold cross-validation	AUC (0.62–0.81)	Stemness features based on RNA	Responder vs. Non-responder	^43^
Predict response	XGBoost	No	Metastatic NSCLC	239	ICI	10-fold cross-validation	AUC (0.72–0.74)	25 variables based on blood immune cell signatures and clinical data	DCB vs. non-DCB	^44^
TME analysis and response prediction	LR	No	ccRCC	172	Anti-PD-(L)1, anti-CTLA4	Hold-out	AUC (up to 0.93)	RNA of selected genes	Responder vs. Non-responder	^45^
Predict response	L2 regularized LR *	Yes	Melanoma, gastric cancer, bladder cancer	729	ICI	LOOCV, Monte Carlo cross-validation	AUC (0.69–0.79 in different datasets)	Network-based biomarkers + gene-based biomarkers + TME-based biomarkers	Response (Responder vs. Non-responder) and OS prediction	^46^
Predict response	CNN *, Attention-based multiple-instance learning	Yes	NSCLC	345	Anti-PD-(L)1	10-fold cross-validation	AUC (up to 0.80)	Radiology, pathology, genomic alternation, TMB	Risk score	^47^
Predict CAR T cell phenotype for immunotherapy response	NN	No	Not mentioned	NA	CAR T therapy	10-fold cross-validation	R squared	Array of signaling motifs of a CAR costimulatory domain + initial CAR T cell count	Quantitively phenotype prediction (cytotoxicity and stemness) from a CAR motif combination	^51^

*machine learning models with a feature selection process, *SVM-RFE* support vector machine recursive feature elimination, *LASSO* least absolute shrinkage and selection operator, *LR* logistic regression, *BLCA* bladder Urothelial Carcinoma, *AUC* area under the curve, *TMB* tumor mutational burden, *RF* random forest, *CNN* convolutional neural network, *DCB* durable clinical benefit, *PFS* progression-free survival, *OS* overall survival, *SKCM* skin cutaneous melanoma, *STAD* stomach adenocarcinoma, *GBM* glioblastoma multiforme, *SVM* support vector machines, *XGBoost* extreme gradient boosting, *NN* neural network, *ICI* immune checkpoint inhibitors, *NMF* non-negative matrix factorization, *NSCLC* non-small-cell lung cancer, *UC* urothelial carcinoma, *RCC* renal cell carcinoma, *KNN* k-nearest neighbors, *GP* Gaussian process, *DT* decision tree, *NB* naïve Bayes, *BCC* basal cell carcinoma, *LOOCV* leave-one-out cross-validation, *GEA* gastroesophageal adenocarcinoma, *PMBC* peripheral blood mononuclear cells, *cfmiRs* circulating cell-free microRNAs, *HNSCC* head and neck squamous cell carcinomas, *MIL* multiple-instance learning, *TCR T*-cell receptor, *MHC* major histocompatibility complex, *RCC* renal cell carcinoma, *UC* urothelial carcinoma, *ccRCC* clear cell renal cell carcinoma, *TME* tumor microenvironment, *NA* not applicable, *CAR* chimeric antigen receptor, *irAE* immune-related adverse events.

Accompanied by the advancement of sequencing technologies, recent studies have focused on developing complex ML models incorporating multi-omics datasets for immunotherapy prediction^42–47^. Compared to single omics approaches, the integration of multiple omics data can provide a more comprehensive scope of tumor profile, from the original cause of tumors (genetic, environmental, or developmental) to the functional consequences^48,49^, and consequently leads to improved performance in immunotherapy response prediction^47^. In a recent approach^50^, researchers integrated RNA-seq data with somatic mutations, copy number alterations and protein expression alterations to comprehensively investigate various subcohorts within TME using a sparse hierarchical clustering model. By employing this method, they have successfully identified distinct subcohorts within the TME, each exhibiting unique responses to different cancer treatments, including immunotherapy. This model holds significant potential in guiding precise decision-making for combination therapy strategies. However, integrating and training multi-omics data, usually accrued from different platforms, is more challenging than training unimodal features. Addressing this challenge, a recent study by Vanguri et al.^47^ developed a dynamic deep attention-based multiple-instance learning model that integrates radiology, pathology, and multiomics data to predict the response of NSCLC patients treated with anti-PD-1/PD-L1 blockade. Comparisons demonstrated that the multimodal approach, integrating these features, enables higher accuracy than unimodal approach in the prediction of immunotherapy response. Notably, their multimodal model can also handle redundant information and missing values in combination with data from different modalities. In addition to ICI prediction, ML models have been utilized to predict the chimeric antigen receptor (CAR) T therapy response. Daniels et al.^51^ developed a DL model to utilize signaling motifs to evaluate the antitumor efficacy of a given CAR. Their DL framework takes the motif sequence of the CAR as the input and propagated the encoded sequence through two CNN layers, a long short-term memory (LSTM) network layer and seven fully connected neural network (FCNN) layers. This approach enables directly prediction of tumor stemness and cytotoxicity based on the motif combinations designed for CAR T cells, thereby guiding the design of the engineering of CAR signaling domains in CAR T therapies.

In our survey, to improve the computational efficiency and reduce noise and complexity of ML models, most studies utilized statistical or ML algorithms, or both to identify a subset of gene markers for model training. ML techniques for gene selection can handle high-dimensional data and identify patterns that may not be apparent through manual inspection or traditional analyses. These ML approaches, including LASSO^29^, random forest (RF)^21,40^, SVM-RFE^22,26^, NMF^28^, and logistic regression (LR)^27^, automatically assess the importance of each gene in relation to the immunotherapy response prediction. Using these extracted biomarkers, various algorithms, including LASSO, LR, RF, XGBoost, naive Bayes (NB), SVM, decision tree (DT) and NN, have demonstrated their ability to accurately predict immunotherapy responses. By focusing on this refined set of features, researchers can also enhance the interpretability and generalizability of the models, fostering a more effective integration of machine learning into the complex landscape of immunotherapy research.

## Employing machine learning as a supplementary tool for the identification of biomarkers in the tumor microenvironment for immunotherapy

**Fig. 3:**
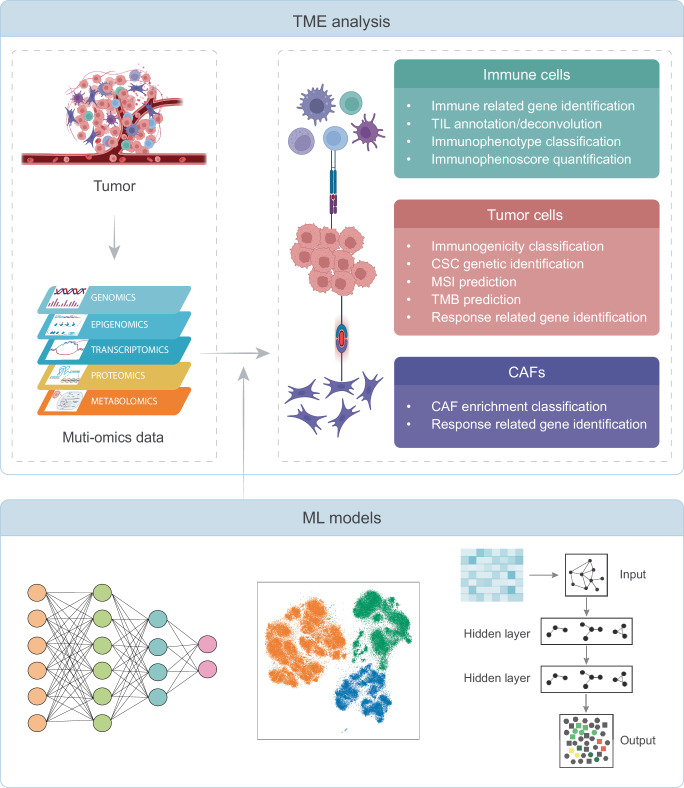
Machine learning offers promising strategies for evaluating tumor microenvironment. Various machine learning models have been developed to effectively identify biomarkers and comprehend the relationship between tumor microenvironment and immunotherapy, including risk, development and treatment. These models aim to improve the efficiency of immunotherapies by providing valuable insights and understanding. ML machine learning, TME tumor microenvironment, TIL tumor-infiltrating lymphocytes, CAF cancer-associated fibroblast, CSC cancer stem-like cell, MSI microsatellite instability, TMB tumor mutational burden.

The TME refers to the intricate cellular landscape surrounding tumors, including immune cells, cancer cells, stroma cells, the inflammatory cytokines and chemokines, metabolites, acidity, cytokines and hypoxia^52^. It plays a critical role in supporting tumorigenesis and progression, and immunotherapy effectiveness^53,54^. Extensive studies have elucidated the complex interactions within TME, driving functions like angiogenesis^55^, metastasis^56^ and immunosuppression^57^. Although obtaining accurate datasets for TME factors such as hypoxia and low pH poses challenges, the integration of tumor omics data and the implementation of ML models have enabled the identification of TME characteristics directly or indirectly associated with cancer immunotherapy (Table 2, Fig. 3).

**Table 2:** Application of machine learning technologies in immunotherapy-related tumor microenvironment analyses

Task	ML Model	Cancer	TME feature type	Input	Output	Ref
Predict MSI status	RF-based model, SVM	Not mentioned	808 cancer-gene panel (DNA, RNA)	54 features based on the sequenced panel	MSI classification: MSI high vs. MSS	^59^
Identify gene target panel to predict TMB and response	LASSO regression	Metastatic melanoma, NSCLC	WES	Somatic mutations	Responders vs. Non-responders and selected mutations	^60^
Identify cancer stem-like signatures	LASSO COX regression	Gastric cancer	RNA	RNA of 2,527 genes	Responders vs. Non-responders and selected stem-like features	^67^
Identify cancer stem-like signatures	Cancer stemness clustering: K-means; Cancer stemness feature selection: LASSO regression, SVM, RFB, XGBoost, LR; Response prediction: TIDE	GBM	RNA	RNA of cancer stemness-associated DEGs	Stemness subtype cluster and selected cancer stemness-associated genes	^68^
Identify CAF signatures	CAF subtype clustering: Consensus clustering; Gene selection: RF, DT, KNN	Melanoma, lung cancer, TNBC	RNA	Prognostic-related RNA data	CAF-subtype clustering and selected subtype-related genes	^70^
Identify CAF signatures	LASSO regression, RF	Melanoma	RNA	DEGs	Responders vs. Non-responders and key CAFs-related DEGs	^71^
Identify gene signatures and immunotherapy response prediction	TME clustering: Hierarchical clustering; Cluster feature selection: LASSO Cox regression, RF; TME cluster classification: SVM, NB, RF, NN; Risk prediction: DT	LUAD	RNA	RNA, clinicopathological traits	TME (risk) cluster classification: low vs. intermediate vs. high and their cluster related gene features	^76^
Identify immune-related genes from protein signatures	Immune-related gene identification: NN Immunotherapy response: DT	Gastric cancer	PPI network data, RNA	PPMI matrix based on PPI network data, RNA	NN: Gene property classification (immune-promoted vs. immune-inhibited); DT: Response prediction (Responders vs. Non-responders)	^77^
Identify TIIClnc	LASSO regularized LR, Boruta, XGBoost, SVM, RF	GBM	RNA	Selected lncRNA	Regulation prediction in immune cell lines and GBM cell lines (upregulated vs. downregulated)	^78^
Identify TIIClnc	LASSO, Ridge, stepwise Cox, CoxBoost, RSF, Enet, plsRcox SuperPC, GBR, survival-SVM	LGG	RNA	Filtered top expressed TIIClnc signatures	Responders vs. Non-responders and selected TIIClnc signatures	^79^
Identify impact of CTLA-4 blockade on antigen-specific, human T-cell responses early between neonates and adults	RF	Healthy donors	Flow cytometry	Frequencies of cytokine producers in the encountered CD4 + T-cell responses	CD4 + T cell classification (neonates vs. adults) after CTLA-4 blockade stimulation	^80^
Predict T cell infiltration	LR, SVM	Colorectal cancer	Histological data, 373 cancer and immune related gene panel from FoundationOne	LR: image-based features SVM: patient’s gene expression profile	T cells and tumor cells co-localized vs. not co-localized	^81^
Predict TIL	Multimodal NN model	Colorectal cancer, breast cancer, lung cancer, pancreatic cancer	RNA, H&E staining images	RNA-seq + Visual texture feature extracted from H&E staining	Proportions of five immune cell types within tumors and total TIL proportions	^82^
Identify epigenomic signatures	RF	LUAD	DNA methylation data	iDMCs	Immunoactivity classification and selected signatures	^83^
TIME deconvolution	nu-SVR-based noise constrained recursive feature selection	Not mentioned	RNA	RNA	Proportions of 22 immune cell types	^84^
Identify tumor-associated metabolism subtypes	Cox regression with LASSO penalty	LUAD	RNA	RNA of 1,426 lipid metabolism genes and 1,638 immune-related genes	Metabolic TME subtype prediction (metabolism vs. immunoactive)	^92^

*MSI* microsatellite instability, *RF* random forest, *SVM* support vector machines, *MSS* microsatellite stable, *LASSO* least absolute shrinkage and selection operator, *TMB* tumor mutational burden, *NSCLC* non-small-cell lung cancer, *WES* whole-exome sequencing, *TIDE* tumor immune dysfunction and exclusion, *GBM* glioblastoma multiforme, *RFB* random forest and Boruta, *CAF* cancer-associated fibroblast, *DT* decision tree, *KNN* k-nearest neighbors, *TNBC* triple-negative breast cancer, *DEG* differentially expressed genes, *LUAD* Lung adenocarcinoma, *PPI* Protein-protein interaction, *PPMI* positive pairwise mutual information, *TIIClnc* tumor-infiltrating immune cell-associated lncRNAs, *GBM* glioblastoma, *RSF* random survival forest, *Enet* elastic network, *plsRcox* partial least squares regression for Cox, *SuperPC* supervised principal components, *GBR* generalized boosted regression, *LGG* low-grade glioma, *TIL* tumor immune infiltration, *iDMCs* immunophenotype-specific differentially methylated CpG sites, *nu-SVR* support vector regression.

### Microsatellite instability and tumor mutational burden

MSI and TMB are FDA-approved biomarkers that predict immunotherapy response. While not directly related to the TME, MSI and TMB reflect genetic alterations occurring within tumor cells. MSI indicates microsatellite length polymorphism due to mismatch repair deficiency, while TMB represents the number of somatic mutations per million bases in the exome region^58^. Tumors with higher MSI or TMB tend to produce neoantigens recognized by the immune system, rendering them more responsive to immunotherapy. Given the high cost of large-scale genomic sequencing, using ML models to assess MSI or TMB based on a panel with limited genes can offer a more cost-effective approach. Zhou et al.^59^ successfully identified a 54-microsatellite-site biomarker using an RF classifier, allowing accurate classification of microsatellite instability-high (MSI-H) and microsatellite stable (MSS) tumors. In a similar vein, Lu et al.^60^ implemented LASSO regression to identify a gene-targeted panel capable of accurately assessing TMB levels. Recently, many deep learning (DL) models have been developed to use whole-slide images (WSIs) to predict MSI^61,62^ and TMB status^63,64^, which enables a more cost-effective means of predicting immunotherapy response without relying on genomic data.

### Cancer stem-like cell

Cancer stem-like cells (CSCs) are a small population of cancer cells that can reconstitute and propagate tumors. They have been implicated in metastasis, relapse, and resistance to cancer therapies^65,66^. Numerous studies have focused on identifying and categorizing CSCs within tumor cell populations. Researchers such as Wei et al.^67^ and Wang et al.^68^ employed LASSO regression to identify stemness features in tumor samples using RNA-seq data. These identified stemness features have shown a strong correlation with the prognosis of immunotherapy and can serve as valuable biomarkers for predicting immunotherapy response.

### Cancer-associated fibroblast

Cancer-associated fibroblasts (CAFs), also a critical component of TME, can modulate cancer metastasis through signaling interactions with cancer cells. They can also influence leukocyte infiltration, drug access and therapy responses^69^. To identify CAF related genes, Wang et al.^70^ applied ML models to classify tumor samples as either CAF-enriched (CAF+) or CAF-absent (CAF−). Their analysis revealed that the CAF− subtype was associated with longer overall survival and higher immune cell infiltration compared to the CAF+ subtype. These findings provide valuable insights for predicting the response to immunotherapy. Similarly, Tian et al.^71^ used LASSO regression to obtain six CAF-related genes that can be used to predict the response to anti-PD-1 therapy in melanoma patients. These studies demonstrate the utility of ML models in elucidating the role of CAFs and their associated genes in immunotherapy response prediction.

### Tumor-infiltrating lymphocyte

TILs are highly specific immunological reactive lymphocytic cell populations that can recognize and kill tumor cells^72^. Their presence is crucial in mediating response to cancer therapy, and a higher abundance of TILs is often associated with better clinical outcomes after immunotherapy^73–75^. Currently, ML models have been broadly implemented to quantify various TIL-based biomarkers for immunotherapy response prediction. These biomarkers encompass RNA-seq data and somatic mutation features^76^, including protein-protein interaction (PPI) networks^77^, tumor-infiltrating immune cell-associated lncRNAs^78,79^, T cell signatures^80,81^, B cell signatures^82^ and immunophenotype-related DNA methylation signatures (iPMS)^83^. A general approach adopted in these models involves clustering tumor samples based on the tumor immune microenvironment, such as immunoactivity, disease stages, and survival outcomes. ML models are then employed to extract significant biomarkers for cluster classification. Subsequently, another ML model was then utilized for predicting immunotherapy response to validate the selected biomarkers for each cluster. Typically, TILs comprise both mononuclear and polymorphonuclear immune cells, including T cells, B cells, natural killer cells, macrophages, neutrophils, dendritic cells, mast cells, eosinophils, and basophils. Accurately assessing the abundance of each immune cell type within tumor tissues is essential for treatment decision-making and evaluating drug response. To this end, ML models have been developed to automatically estimate the abundance of these immune cells^82,84–87^, enabling precise deconvolution. In a recent study, Fernández et al.^84^ used their deconvolved proportions of 22 immune cells as the input feature, which could accurately predict the response of patients treated with ICI therapy.

### Metabolism

Metabolism refers to the changes observed in cellular metabolic pathways in tumor cells. Typically, oncogenic transformation can induce cancer cells to adopt a well-characterized metabolic phenotype that can profoundly influence the TME^88^. Increasing evidence has highlighted the role of metabolism in tumor immunosuppressive responses and resistance to immunotherapy^89,90^. For instance, tumor cells can alter metabolism by increasing glucose uptake and fermentation of glucose to lactate, promoting tumor growth, survival, proliferation, and long-term maintenance^91^. To improve immunotherapy efficacy, researchers have proposed employing ML models to identify metabolic TME subtype that respond favorably to immunotherapy. Ge et al.^92^ conducted an analysis of lipid metabolism genes and immune-related genes of lung adenocarcinoma (LUAD) patients and identified two distinct subtypes, namely “metabolism phenotype” and “immunoactive phenotype”, using Cox regression. The “metabolism subtype” exhibited reduced sensitivity and poorer prognosis to immunotherapy. The identified metabolic features hold promise as potential biomarkers to predict immunotherapy response.

### Neoantigen

Neoantigens are novel peptides that form in tumor cells due to certain somatic mutations. Neoantigens have the potential to be recognized by immune cells, triggering immune responses against tumor cells^93,94^. Immunogenic neoantigens have been identified as crucial for developing personalized neoantigen-targeted cancer immunotherapies^95,96^, including vaccines and adoptive T-cell therapies^94^. However, the process of neoantigen discovery and validation remains a daunting question that must be addressed before neoantigen-based immunotherapies can become prominent in cancer treatment^97^. For example, many tumor peptides lack immunogenicity, highlighting the importance and complexity of accurately identifying which neoantigens can effectively stimulate immune cell responses.

**Fig. 4:**
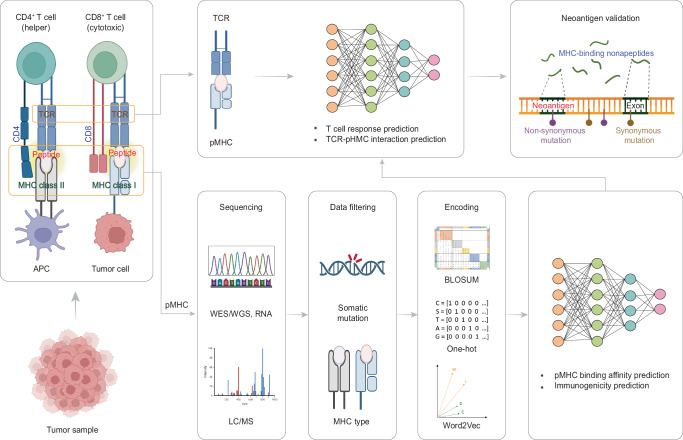
Identification of tumor neoantigens using machine learning models. The identification of tumor neoantigens involves the elution of MHC epitopes from tumor cells and the extraction of somatic mutations from sequencing data. Machine learning algorithms are then processed to model the binding affinity between mutant peptides and MHC proteins, allowing for the prediction of candidate neoantigens. To improve performance, some models incorporate TCR sequencing data to screen for candidate neoantigens with high proportions that interact with TCR and induce T cell responses. MHC major histocompatibility complex, TCR T-cell receptor, APC antigen-presenting cell, pMHC peptide-MHC, WES whole-exome sequencing, WGS whole-genome sequencing, LC/MS liquid chromatography/mass spectrometry.

Recently, novel pipelines and state-of-the-art ML algorithms have been developed to identify T-cell neoantigens through major histocompatibility complex (MHC) class I and II presentations (Table 3, Fig. 4). Pipelines utilize genomics data, usually derived from whole-genome sequencing (WGS) or whole-exome sequencing (WES), obtained from tumor samples to infer the mutated peptides based on the somatic non-synonymous SNVs. To facilitate neoantigen prediction, researchers have conducted The Immune Epitope Database (IEDB), which provides experimentally characterized T cell epitopes and a comprehensive set of MHC-binding and MHC eluted ligand (EL) data for humans^98^. These resources significantly enhance the convenience and accuracy of neoantigen prediction. Based on our review, the majority of ML models focus on identifying class I MHC alleles, which have the ability to bind peptides derived from intracellular proteins and present them on the cell surface to CD8 + T cells. Some studies employ ML models to predict neoantigens by estimating the binding affinity between a given mutated peptide and a class I MHC molecule, known as peptide-MHC (pMHC) binding affinity^99–106^. These models can be categorized into two groups based on their output. The first group predicts a score representing the relative binding affinity between a peptide and MHC^99–103^. Among these models, NetMHC^99^ and NetMHCpan^100^ used the FCNN framework. While NetMHC was trained solely on binding affinity datasets and can predict peptide binding to specific MHC alleles, NetMHCpan integrated information from both binding affinity data and mass spectrometry (MS) EL data, allowing it to predict binding for a wider range of MHC molecules with high accuracy. Different from NetMHC and NetMHCpan, MHCflurry^101^ added two one-dimensional convolutional layers before fully connected layers, resulting in higher accuracy. EDGE^102^, on the other hand, used three peptide-extrinsic features (RNA abundance, flanking sequence, per-gene coefficients) captured from MS data as the input, propagating them into three locally connected layers respectively before merging them into fully connected layers for binding affinity prediction. This approach extracts more information than using a single input, resulting in higher positive predictive values (PPV) compared to benchmarked models. Another model, MHCRoBERTa^103^, built a transfer learning model by pre-training on the UniprotKB/Swiss-Prot dataset and fine-tuning on IEDB dataset. This strategy allows the model to maintain high accuracy and efficiency simultaneously. The second form of binding affinity prediction in these models involves providing a binary classification result to determine whether a given peptide is a binder^104–106^. In most studies, a threshold of <500 nM of the IC50 value is used to define candidate peptides that are likely to bind to MHC. Notably, to improve performance, ForestMHC^105^ considered six different sequence-related features and their combinations as input features to select the optimal feature subset. Similarly, Anthem^106^ collected five published sequence scoring functions that can calculate a binding probability based on sequence information. These scoring functions, along with their combinations, were used as input features to select the optimal subset of scoring functions for binder classification. Considering the distinct advantages and limitations of each binding affinity model, Gartner et al.^107^ built a random forest-based model that integrates known class I candidate human tumor neoantigens predicted by other models and next-generation sequencing (NGS) data from individuals with metastatic cancers. This model can rank the candidate neoantigens, providing a ranked list that can serve as therapeutic targets and facilitate studies aimed at developing more effective immunotherapies. Recently, increasing evidence indicates that CD4 + T cells can recognize cancer-specific antigens and control tumor growth. As a result, MHC class II neoantigen prediction has become important in immunotherapies like vaccine design and targeted therapy development. However, unlike MHC class I molecules that are highly specific and bind a limited set of peptides of a narrow length distribution^108^, MHC class II molecules are highly polymorphic and the size of the peptides presented are promiscuous^109^, making it more challenging for neoantigen prediction. In response to this challenge, several models have been established to predict the binding affinity of pMHC class II complexes^110–114^. Compared with class I binding affinity prediction models, the MHC class II prediction models were generally trained on more complicated datasets, such as the IEDB MHC class II cell surface receptor (IEDB MHC-DR) restricted peptide-binding dataset. In particular, MHC class II prediction models need to consider longer or even variable peptide lengths as their inputs.

**Table 3:** Application of machine learning technologies in neoantigen and immunogenicity prediction

Model	Task	ML Model	Encoding method	MHC class	Ref
NetMHC-4.0	Predict binding affinity	NN	BLOSUM	class I	^99^
NetMHCpan-4.0	Predict binding affinity	NN	BLOSUM	class I	^100^
MHCflurry	Predict binding affinity	A deep learning model includes locally connected 1D-CNN and FCNN	BLOSUM	class I	^101^
EDGE	Predict binding affinity	NN	One-hot	class I	^102^
MHCRoBERTa	Predict binding affinity	BPE	Byte pair	class I	^103^
Vang et al.	Predict binding affinity	CNN	Skip-gram	class I	^104^
ForestMHC	Predict binding affinity	RF	NA	class I	^105^
Anthem	Predict binding affinity	NB, XGBoost, LR, NN, SVM, DT, RF	BLOSUM	class I	^106^
Gartner et al.	Rank binding affinity	RF	NA	class I	^107^
NN-align	Predict binding affinity	NN	BLOSUM	class II	^110^
MixMHC2pred	Predict binding affinity	Linear regression	BLOSUM	class II	^111^
NeonMHC2	Predict binding affinity	CNN	One-hot	class II	^112^
NetMHCIIpan	Predict binding affinity	NN	BLOSUM	class II	^113^
MARIA	Predict binding affinity	LSTM	One-hot	class II	^114^
Neopepsee	Predict immunogenicity	LNB, GNB, RF, SVM	NA	class I	^115^
DeepNetBim	Predict immunogenicity	A deep learning model includes CNN and attention module	BLOSUM	class I	^116^
DeepHLApan	Predict immunogenicity	BiGRU + attention	One-hot	class I	^117^
Seq2Neo	Predict immunogenicity	CNN	One-hot	class I	^118^
TCIA	Predict cancer immunogenicity	RF	NA	class I, class II, non-classical	^120^
Besser et al.	Predict CD8 + T cell response	RF	NA	class I	^121^
iTTCA-Hybrid	Predict CD8 + T cell response	SVM, RF	NA	class I	^123^
DLpTCR	Predict TCR-pMHC interaction	A multimodal model based on FCNN, LeNet-5, ResNet-20	One-hot, PCA, PCP	class I	^124^
pMTnet	Predict TCR-pMHC interaction	AE + LSTM + NN	BLOSUM	class I	^125^

*NN* neural network, *BPE* byte pair encoding, *CNN* convolutional neural network, *FCNN* fully connected neural network, *RF* random forest, *NA* not applicable, *NB* naïve Bayes, *LR* logistic regression, *SVM* support vector machines, *DT* decision tree, *LSTM* long short-term memory, *LNB* locally weighted naïve Bayes, *GNB* Gaussian naïve Bayes, *BiGRU* bidirectional Gated Recurrent Unit, *pMHC* peptide-MHC, *PCA* principal component analysis, *PCP* physicochemical properties, *AE* autoencoder.

Typically, identifying binding affinity between MHC and peptides alone is insufficient for accurate neoantigen predictions with high confidence. To overcome the limitation, some studies have focused on assessing the immunogenicity of the predicted binding molecules^115–118^. Immunogenicity refers to the ability of protein products to provoke an immune response, and it depends on several factors, including protein expression, peptide-MHC binding affinity and stability, peptide competition for MHC binding, and more^94,119^. Among these models, DeepHLApan^117^ designed a multi-task neural network model consisting of three layers of bidirectional Gated Recurrent Unit (BiGRU) with an attention layer. This model can simultaneously predict the binding affinity and the immunogenicity. Similar to DeepHLApan, DeepNetBim^116^ used a CNN model with an attention layer to predict binding affinity and binary immunogenic categories. In comparison to DeepHLApan, DeepNetBim incorporated an additional layer to merge the two independent outputs together, namely the binding affinity and the binary immunogenic category, in order to calibrate the final immunogenicity prediction. Seq2Neo^118^ took a different approach by developing an end-to-end software that directly utilize raw sequencing data (WES/WGS, RNA) in FASTQ, SAM and BAM formats to predict the immunogenicity directly through a CNN-based model. In contrast, Charoentong et al.^120^ did not focus on developing a state-of-the-art DL model like most approaches. Instead, they designed a comprehensive biomarker consisting of 127 features, including somatic mutation features, class-I and class-II MHCs, immune inhibitory and stimulatory genes, adaptive immunity cells and innate immunity cells from integrated WES, RNA-seq and clinical data. Their results demonstrated that proper feature extraction could achieve a high accuracy for tumor immunogenicity prediction using only an RF classifier. In addition to assessing the immunogenicity of the predicted binding molecules, some studies have explored the integration of TCR sequence to predict the likelihood of peptide-TCR interaction for neoantigen prediction. Besser et al.^121^ proposed using CD8 + T cell responses as the task of their models to detect neoantigens. To accomplish this, they employed an additional step in their ML models, training them on the Tantigen dataset^122^, a comprehensive database of tumor T cell antigens. Through this step, they were able to learn the changes in key parameters and features associated with T cell response, enabling them to predict whether a given MHC class I peptide was positive for inducing CD8 + T cell response. Likewise, iTTCA-Hybrid^123^ utilized the tumor T cell antigen dataset from Tantigen^122^ and non-tumor T cell antigen dataset from IEDB^98^ to train an ensemble model capable of classifying tumor and non-tumor T cell antigens. More recently, DLpTCR^124^ and pMTnet^125^ suggested that assessing the propensity of CD8 + TCR to recognize the pMHC complex is crucial for neoantigen prediction, as most in silico predicted antigen peptides fail to elicit immune responses in vivo. Both models take peptide and TCR sequences as input data, and their output is a binary classification of whether the TCR-pMHC has an interaction. To achieve better performance, DLpTCR^124^ designed an ensemble strategy based on three deep learning models: FCNN, LeNet-5 and ResNet. On the other hand, pMTnet^125^ utilized an autoencoder and an LSTM network to obtain the hidden encoding of the TCR sequence and peptide sequence, respectively. These encodings were then fed into an FCNN classifier for final prediction.

It is worth noting that peptide sequence encoding plays a crucial role in neoantigen prediction. Two commonly employed methods for encoding are one-hot encoding and BLOcks SUbstitution Matrix (BLOSUM) encoding (Table 3). Among them, BLOSUM is more prevalent as it offers insights into the homologies between protein sequences. In addition, personalized sequencing encoding techniques utilizing ML algorithms have also gained popularity. These include byte pair encoding^103^, skip-gram encoding^104^, principal component analysis (PCA) encoding^124^ and physicochemical properties (PCP) encoding^124^.

In conclusion, ML has emerged as a promising approach for evaluating TME, identifying TME related biomarkers and unraveling the intricate relationship between TME and immunotherapy. The biomarkers derived from ML approaches hold great potential for predicting clinical outcomes of immunotherapy and enhancing personalized immunotherapy strategies, thereby facilitating the advancement and wider application of immunotherapy in cancer treatment.

## Challenges and opportunities

Despite the extensive application of ML in immunotherapy studies, several challenges remain to be addressed. These challenges pertain to gaining a mechanistic understanding of how immunotherapies target and eradicate tumor cells^126^ and the neoantigens that can be recognized by immune cells^127^. Whether and how ML models prompt the progression of immunotherapy will depend on how these challenges, as discussed below, are met in the future.

### Insufficient amount of available data

Immunotherapy has emerged as a promising cancer treatment, driving numerous clinical trials worldwide^128^. Nevertheless, current clinical trials have primarily focused on PD-1/PD-L1 therapy, result in limited data for other treatment like CTLA-4 and CAR T therapy (Table 1). This data scarcity poses a significant barrier for developing ML models, particularly DL models that require substantial training data to avoid overfitting and enhance model performance^129^. To mitigate the limitations, the generation of pseudo databases has emerged as a potential solution. State-of-the-art generative models, such as generative adversarial network (GAN)^130^ and diffusion models^131^, have shown promise in computer vision and can generate synthetic data to supplement training datasets, mitigating overfitting issues. Likewise, Sové et al.^132^ developed a model using an ML approach to capture interpatient diversity in clinical trials, allowing the simulation of virtual patients. By leveraging these virtual patients, it becomes possible to mimic a virtual clinical trial scenario to quantitatively assess the efficacy of ICI treatments in a controlled environment.

### Multi-omics data integration and analysis

The advent of multi-omics technologies has revolutionized our understanding of the biological mechanisms of driving immunotherapy. However, analyzing these large multi-omics data, particularly those from single-cell-based^133^ and spatial-based^134^ technologies, has brought new computational challenges. One challenge is the batch effects, resulting from diverse platforms used for data generation. To ensure accurate downstream analyses, removing platform-specific noise is crucial. Recently, ML models, particularly joint dimension reduction algorithms such as negative matrix factorization (NMF), PCA, singular value decomposition (SVD), canonical correlation analysis (CCA), have emerged as powerful tools for encoding data from diverse platforms into a shared latent space, thereby enabling effective batch effect removal^135^. Additionally, the training data often exhibit distinct statistical modalities. To tackle this challenge, multimodal learning with specialized modelling strategies has gained attention for integrating diverse data modalities, such as medical imaging and genomics^41,47^. By harnessing the strengths of multiple modalities, multimodal learning models offer the potential to address immunotherapy-related questions.

### Meta-analysis

In the field of immunotherapy response prediction, the definitions of “response” vary across studies. For example, Vanguri et al.^47^ and Chowell et al.^42^ employed Response Evaluation Criteria in Solid Tumors (RECIST)^136^ as their criterion for defining response, whereas Filipski et al.^38^ utilized survival (defined as the time from start of ICI treatment to date of decease) to characterize response. The disparate use of these distinct criteria underscores the considerable variability in how the concept of “response” is operationalized across studies, posing a challenge to the synthesis of studies and the establishment of a standardized framework for meta-analysis. Standardization the definition and harmonization data are necessary to achieve a consensus on common criteria or thresholds for defining immunotherapy response.

### Neoantigen prediction

With ongoing developments of new algorithms, the field of cancer neoantigen identification holds promise for immunotherapies^94^. Given the uniqueness of the neoantigen landscape to each individual, the accurate targeting of neoantigens establishes a solid foundation for conducting systematic studies in precision medicine and providing clinical decision support for cancer immunotherapy. Computational models, especially ML algorithms, are commonly used for immunogenic neoantigen prediction. However, comparative studies have revealed that, thus far, none of the existing studies have achieved accurate identification of immunogenic neoantigens^127^. Factors such as tumor heterogeneity, diversity within the TCR repertoire, and the absence of true labeled data contribute to this inaccuracy. Future studies should focus on developing more comprehensive models integrating both pMHCs and TCR sequencing data to improve predictive performance of neoantigen identification. It is worth noting that certain studies have explored targeting tumor-specific gene fusion^137^ and MHC gene loss of heterozygosity (LOH)^138^ to improve immune recognition in neoantigen identification. Incorporating these factors could augment neoantigen predictions and contribute to higher accuracy in future studies.

### Model generalizability and interpretability

While numerous ML models have been developed for immunotherapy response prediction, they often struggle to adapt well to unseen data. Their performance on new data is often moderate or deficient, indicating a lack of generalizability. Moreover, these models typically employ ML or statistical approaches to select marker genes. However, the selected marker genes vary between studies and may have limited effectiveness within specific datasets. To address these challenges, recent studies have employed transfer learning algorithms for immunotherapy response prediction. By leveraging pre-trained models and applying them to train on new, similar datasets^39^, this approach can enhance the efficiency and robustness^139^. In addition, the interpretability of ML models in immunotherapy remains a persistent concern, ML algorithms often function as black boxes, making it difficult to understand the decision-making process and the underlying biological rationale behind their predictions. To improve the generalizability, researchers are exploring feature insights and interactions through explainable AI (XAI) models^140^. XAI approaches can provide global and local explanations, enabling a deeper understanding of predictions and facilitating effective fine-tuning on new data.

### Models in handling continual incremental datasets with real-time adaptation

In our studies we reviewed, almost all models applied for immunotherapy analyses are traditional batch learning approaches. These methods utilized entire datasets simultaneously for training, deploying the trained model for inference without frequent updates. However, they usually encounter high retraining cost when adapting to new training data^141^. With the growing of clinical and genomics data during the patient treatment, there is a need to develop models with the capacity to conduct incremental datasets and adapt in real-time to new information. Online learning emerges as a scalable and efficient approach that learn to continuously updates the model based on feedback on its decisions in the form of a sequence of examples^141–143^, demonstrating premium performance in clinical applications^144^. This approach holds the potential to significantly assist clinicians via providing diagnoses or making management decisions.

### Clinical translation

While numerous ML models have been developed for predicting immunotherapy outcomes, our review reveals that almost none of these models have undergone clinical testing. Furthermore, contemporary ML-based clinical decision support systems, such as IBM Watson Health^145^ and Google DeepMind Health^146^, encounter obstacles hindering the smooth transition of models from research settings to standard clinical practice. This discrepancy underscores the critical necessity for rigorous clinical validation to evaluate the real-world efficacy and reliability of these predictive models. The complexity of the immune system, the dynamic nature of immunological responses, the lack of data quality and standardization, and the absence of highly reliable biomarkers all contribute to the challenges impacting the performance of these models. Conducting comprehensive clinical trials and validation studies is crucial to bridging the gap between theoretical concepts and practical applications in the field of immunotherapy.

### Opportunities

Despite the limited number of databases, there are still a growing number of resources available for immunotherapy research. The Cancer Genome Atlas (TCGA)^147^ is a prevalent curated database containing genomic, epigenomic, transcriptomic, proteomic and whole slide imaging data across 33 cancer types. Among them, a significant number of patients were treated with immunotherapy, and these samples have been widely used in training ML models as reviewed in this study. In addition, the medical images (MRI, CT, digital histopathology, etc.) of some of these patients can be downloaded from The Cancer Imaging Archive (TCIA)^148^ database, enabling the multi-modality analysis of immunotherapy studies. Tumor Immunotherapy Gene Expression Resource (TIGER)^149^ and ICBatlas^150^ are comprehensive resources for integrative analysis of the transcriptome profiles related to tumor immunology. The Cancer Immunome Atlas^120^ is a web-accessible database that characterizes the intratumoral immune landscapes and the cancer antigenomes of 20 solid cancers. This database has also developed an immunophenoscore to quantify tumor immunogenicity from genomic features, which helps inform cancer immunotherapy and facilitate the development of precision immuno-oncology. To ensure safe cancer treatment, Wang et al.^151^ developed an irAE data resource consisting of a total of 893 irAEs. They also performed comparative analyses on these irAEs, making it more intuitive to identify and understand how off-targets of ICIs are involved in irAEs. In addition to clinical resources, there are datasets available for other immunotherapy-related collections. IEDB^98^ and Tantigen^122^ provide a comprehensive set of data related to antibody, B and T cell epitopes for humans, along with tools to assist in the prediction and analysis of neoantigens for immunotherapy. In summary, these resources and databases have facilitated the generation of new research tools, diagnostic techniques, vaccines and therapeutics that were previously used in immunotherapy studies.

## Conclusions

Immunotherapy holds promise for cancer treatment, but the rapid accumulation of immunotherapy-related data has raised challenges. This review summarizes the use of ML approaches in addressing these challenges. Conventional ML algorithms (LR, RF, SVM, LASSO, XGBoost) have demonstrated their versatility in handling various omics datasets, including mutations, CNVs, methylation profiles, and expression profiles, to predict immunotherapy responses. ML models also analyze TME to identify biomarkers and subcohorts with distinct immunotherapy responses. Unsupervised clustering algorithms are typically utilized for subcohort identification, while LASSO regression is employed to identify subcohort biomarkers. Notably, DL approaches are extensively implemented for handling the sequencing data in neoantigen prediction. Natural language processing-related models, including word-to-vector models, are broadly used for sequence encoding, whereas recurrent neural networks-based models or transformers are commonly utilized for task training. Moreover, we highlight the prevailing challenges, emphasizing the need for ML models to handle multi-modal data to facilitate the rapid accumulation of imaging and omics data. Ultimately, this review aims to inspire cutting-edge ML research in maximizing the potential of immunotherapies.

### Reporting summary

Further information on research design is available in the Nature Research Reporting Summary linked to this article.

### Supplementary information

Reporting Summary

## Data Availability

The authors hereby declare that all pertinent data has already been displayed within the article. Additional data can be accessed upon request to the corresponding author.
